# Neural Network-Based Calculator for Rat Glomerular Filtration Rate

**DOI:** 10.3390/biomedicines10030610

**Published:** 2022-03-05

**Authors:** Óscar J. Pellicer-Valero, Giampiero A. Massaro, Alfredo G. Casanova, María Paniagua-Sancho, Isabel Fuentes-Calvo, Mykola Harvat, José D. Martín-Guerrero, Carlos Martínez-Salgado, Francisco J. López-Hernández

**Affiliations:** 1 Intelligent Data Analysis Laboratory (IDAL), Department Electronic Engineering, School of Engineering (ETSE-UV), Universitat de València, 46100 Valencia, Spain; oscar.pellicer@uv.es (Ó.J.P.-V.); mykola.harvat@uv.es (M.H.); jose.d.martin@uv.es (J.D.M.-G.); 2 Institute of Biomedical Research of Salamanca, 37007 Salamanca, Spain; giampieroandrea.massaro@usal.es (G.A.M.); alfredogcp@usal.es (A.G.C.); meripani@usal.es (M.P.-S.); ifc@usal.es (I.F.-C.); carlosms@usal.es (C.M.-S.); 3 Departmento de Fisiología y Farmacología, Universidad de Salamanca, 37007 Salamanca, Spain; 4 Fundación Instituto de Estudios de Ciencias de la Salud de Castilla y León, 42002 Soria, Spain; 5 Group of Translational Research on Renal and Cardiovascular Diseases (TRECARD), 37007 Salamanca, Spain; 6 National Network for Kidney Research REDINREN, RD016/0009/0025, Instituto de Salud Carlos III, 28029 Madrid, Spain; 7 Disease and Theranostic Modelling Consortium (DisMOD), 37007 Salamanca, Spain; 8 Group of Biomedical Research on Critical Care (BioCritic), 47003 Valladolid, Spain

**Keywords:** rat glomerular filtration rate, creatinine clearance, calculator, neural network, machine learning

## Abstract

Glomerular filtration is a pivotal process of renal physiology, and its alterations are a central pathological event in acute kidney injury and chronic kidney disease. Creatinine clearance (ClCr), a standard method for glomerular filtration rate (GFR) measurement, requires a long and tedious procedure of timed (usually 24 h) urine collection. We have developed a neural network (NN)-based calculator of rat ClCr from plasma creatinine (pCr) and body weight. For this purpose, matched pCr, weight, and ClCr trios from our historical records on male Wistar rats were used. When evaluated on the training (1165 trios), validation (389), and test sets (660), the model committed an average prediction error of 0.196, 0.178, and 0.203 mL/min and had a correlation coefficient of 0.863, 0.902, and 0.856, respectively. More importantly, for all datasets, the NN seemed especially effective at comparing ClCr among groups within individual experiments, providing results that were often more congruent than those measured experimentally. ACLARA, a friendly interface for this calculator, has been made publicly available to ease and expedite experimental procedures and to enhance animal welfare in alignment with the 3Rs principles by avoiding unnecessary stressing metabolic caging for individual urine collection.

## 1. Introduction

Alterations in GFR are a hallmark of many renal ailments, including acute kidney injury (AKI) [1] and chronic kidney disease (CKD) [2,3], and a gold standard diagnostic parameter [4,5,6]. Methods to measure GFR are based on the clearance of specific probe molecules from the blood. These must be cleared solely by renal excretion, not metabolized, freely filtered at the glomerulus, or secreted or reabsorbed by the tubule, and must not interfere with the GFR [7,8]. Different exogenous molecules with these characteristics have been used [8,9,10], but the clearance of inulin (ClIn), a plant fructan oligosaccharide, is still the gold standard GFR measurement method [7,11,12,13].

These clearance methods are overtly inconvenient for routine clinical practice [14,15] due to the time-consuming and costly procedures, such as the need for exogenous probe administration, timed urine and blood collection and analysis, and the involvement of radioactivity [9]. For these reasons, GFR measurement turned to endogenous probes [16], the most used of which is creatinine (Cr), a metabolite of phosphocreatine and a waste product of muscle activity. Creatinine clearance (ClCr) is not the ideal method for GFR measurement either, because Cr is also secreted in the tubule and, in some circumstances, can also be cleared from the organism with the feces [17]. In fact, 10–40% of the creatinine found in the urine is secreted in normal individuals [18], which leads to an overestimation of the GFR when measured as ClCr, compared to ClIn [19]. Yet endogenous ClCr, introduced in 1937 [20], avoids probe administration and the use of radioactivity because Cr can be measured with a colorimetric reaction known as Jaffe’s method [7,21,22], or with enzymatic assays [23]. Its reduced complexity, cost, and risk rapidly led to the adoption of ClCr [7], which still requires timed urine sampling (usually 24-h collection).

Serum creatinine concentration (sCr) is a gross and inverse reflection of ClCr and, thus, of the GFR because it is produced in the muscle at a more or less constant average daily rate. Variations in sCr are therefore mostly derived from changes in ClCr. Accordingly, with all its limitations [24,25], sCr was progressively adopted as the election proxy to estimate GFR, especially after methods for automatic analysis were developed by the 1960s [21,25]. Still, sCr is dependent on muscle mass, race, and other factors; thus, algorithms have been developed to estimate GFR from sCr and personal and body habitus data [9]. In 1976, the Schwartz formula (adjusted by height) [26] and the Cockroft–Gault equation (adjusted by age and weight) [27] were published. Over 25 formulas have been developed thereafter [28], most notably including the Modification of Diet in Renal Disease equation (MDRD) [29] and the Chronic Kidney Disease Epidemiology Collaboration (CKD-EPI) group equation [30], both adjusted by age, sex, and ethnicity. Due to its reasonably high accuracy and practicality, estimated GFR has substituted measured GFR (i.e., clearance methods) in most clinical situations [4]. Recently, a novel approach has been proposed to efficiently estimate GFR from sCr through a numerical algorithm based on bivariate numerical modeling [31].

Similarly, in experimental research with animals, ClIn [32,33] is the preferred method for GFR assessment, but endogenous ClCr [34,35] is most widely used due to reduced complexity. Still, individual, laborious 24-hour urine collection is necessary. A transcutaneous exogenous fluorescence-labeled sinistrin clearance (ClSin) method has been developed which avoids individual housing and allows longitudinal studies with minimal invasiveness [36]. However, ClSin is also laborious and technically more demanding than traditional clearance techniques. Due to simplicity, sCr is again the most frequent surrogate for GFR.

In this study we developed and experimentally validated a neural network-based calculator of ClCr for rats from a single analyte (i.e., sCr) and body weight data, useful in both healthy and disease conditions. This calculator may be used in many acute and chronic pathophysiological circumstances to prevent long, tedious, and costly procedures and avoid the stressful conditions for experimental animals associated with ClCr measurement.

## 2. Materials and Methods

### 2.1. Data Mining and Database Generation

**Figure 1:**
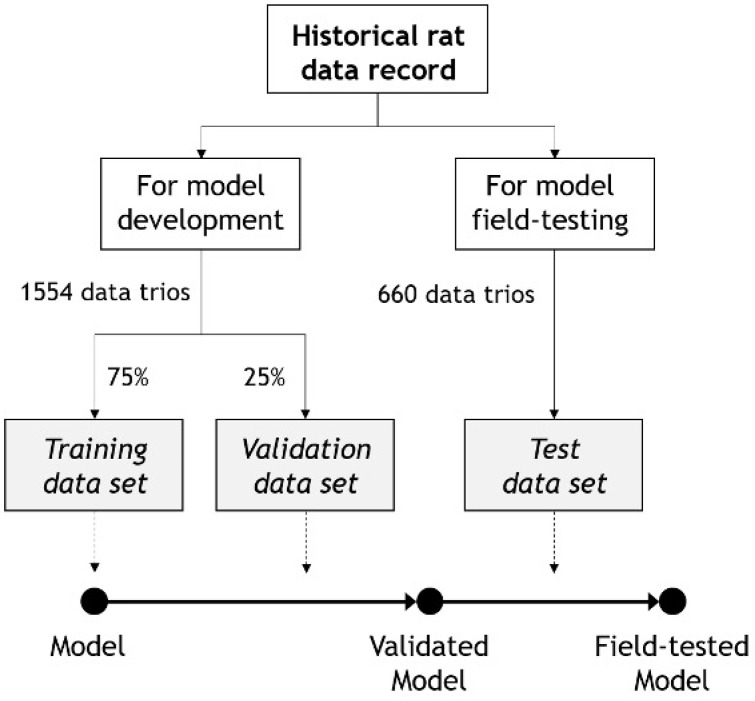
Summary of the datasets used for model development and field testing. Data trios were composed of matched pCr, ClCr, and body weight data.

To train the model, 1554 data trios of measured ClCr (mClCr), pCr, and body weight were collected from the historical records of our laboratory. These datasets came from published and unpublished experiments with AKI and CKD models (and their healthy, normal controls) (Figure 1 and Table 1). A random subset with 75% of the data trios (1165) was used for model training (i.e., the training set), and the remaining 25% (389) was used for validation (i.e., the validation set), which was utilized for making design decisions (e.g., model selection, preprocessing pipeline design, etc.). Once the final model was obtained, it was field-tested against a third dataset with 660 data trios (i.e., the test set) from rats and experiments different from those used for training and validation. In all cases, the data arose from male Wistar rats, with no exclusion criteria. ClCr had been measured as we previously described [37,38]. Briefly, ClCr had been determined with the following formula: ClCr = UF24h × uCr/pCr, where UF24h is the volume of urine collected in 24 h, pCr is the plasma concentration of creatinine, and uCr is the urinary concentration of creatinine. For individual urine collection, rats had been allocated in metabolic cages and allowed free access to water and regular chow. Blood samples had been collected from a small incision in the tail and immediately centrifuged to obtain the plasma, which was frozen at −80 °C until use. Both pCr and uCr had been measured with a commercial colorimetric assay based on Jaffe’s method (Quantichrom Creatinine Assay Kits, BioAssay Systems, Hayward, CA, USA) following the manufacturer’s instructions. A database was generated with rat identifiers, experimental model, ClCr, pCr, and body weight values.

### 2.2. Algorithm Description

Linear regression (LR), random forest (RF), and feedforward neural network (FFNN) [43,44] models were evaluated comparatively. Among them, LR is the simplest algorithm as it can only learn independent linear relationships between each input feature and the output. Conversely, RF is a non-linear model able to capture complex relationships among input and output features as it uses the compound prediction from many (e.g., 100) decision trees to perform regression. Finally, FFNNs are connectionist models that allow for fitting non-linear relationships by stacking many LR units (i.e., neurons) with non-linear activation functions in between, similar to how simple biological neurons combine to form a complex net: the brain. For the LR and RF models, the Scikit-learn library (version 0.24.1) [45] was employed, while for the FFNN, TensorFlow (version 2.1.0) [46] was used.

### 2.3. Data Preprocessing

The relationship between pCr and mClCr was linearized by logarithmic transformation (Equation (1) and Supplementary Figure S1) to help the ML algorithms better capture the relationship. The input features (pCr and weight) were then standardized by Equation (2), which centers the data distribution around zero and scales it to unit variance. Standardization is required by LR, and it is helpful for training other ML models too since it equalizes the relative importance of the features.

(1) $$f\left(x\right)=\log\left(\frac{1}{x}\right)$$

(2) $$\tilde{x}=\frac{x-mean\left(x\right)}{std\left(x\right)}$$

### 2.4. Neural Network Training, Validation, and Testing

For FFNN training, the parameters, i.e., weights, minimizing the model’s prediction error, i.e., the mean squared error (MSE, Equation (3)), between *eClCr* and *mClCr* in the training set were found by the stochastic gradient descent (SGD) [47] iterative optimizer.

(3) $$MSE\left(eCl_{Cr},\;mCl_{Cr}\right)=mean\left({\left(eCl_{Cr}-mCl_{Cr}\right)}^{2}\right)$$

Model performance was mainly assessed with the mean absolute error (MAE) and the Pearson product-moment correlation coefficient (Equations (4) and (5), respectively), although p10 and p30 metrics were also included for completeness. The MAE represents the absolute error (in mL/min) that is committed on average in every prediction. The correlation coefficient measures the strength of the relationship between *mCl_Cr* and *eCl_Cr* with a value between 0 and 1, where 1 stands for a perfect prediction performance (i.e., *eClCr* = *mClCr* for all data trios). Finally, p10 and p30 represent the fraction of predictions (*eCl_Cr*) that fall within 10% and 30% of the *mCl_Cr*, respectively.

(4) $$MAE\left(eCl_{Cr},\;mCl_{Cr}\right)=mean\left(\left|eCl_{Cr}-mCl_{Cr}\right|\right)$$

(5) $$corr\left(eCl_{Cr},\;mCl_{Cr}\right)=\frac{cov\left(eCl_{Cr},\;mCl_{Cr}\right)}{std\left(\;mCl_{Cr}\right)\cdot std\left(eCl_{Cr}\right)}$$

To avoid potential overfitting, all engineering decisions (FFNN architecture, number of layers, neurons per layer, activation functions, etc.) were made based on the validation MAE and correlation coefficient.

Once the model (NN architecture + weights) was finalized, it was challenged with the test set as a proxy for field performance.

## 3. Results

### 3.1. Model Development, Evaluation, and Validation

With the objective of developing a machine learning (ML) model (i.e., a calculator) to estimate rat ClCr (i.e., estimated ClCr; eClCr) from plasma creatinine concentration (pCr) and body weight, three ML algorithms were assessed and compared: LR, RF, and FFNN. Table 2 shows the performance metrics for the three models after training and tuning them. The FFNN outperformed the LR and RF models on the validation set and was therefore selected for further testing. Values of eClCr were compared to the measured (i.e., experimental) values of ClCr (mClCr).

The optimal FFNN architecture was found to have three intermediate (hidden) layers with 30, 20, and 10 neurons respectively, with the hyperbolic tangent as an activation function and using the Adam SGD optimizer with a learning rate of 0.001 and a batch size of 400 with MSE as the loss function. With the aid of the early stopping technique, training was stopped after 200 SGD iterations without improvement of the validation loss. The characteristics of the final FFNN architecture are shown in Supplementary Figure S2 and a decision surface map for the FFNN is shown in Supplementary Figure S3. Agreement between eClCr and mClCr was also studied in the test set by means of a Bland–Altman plot (Supplementary Figure S4).

**Figure 2:**
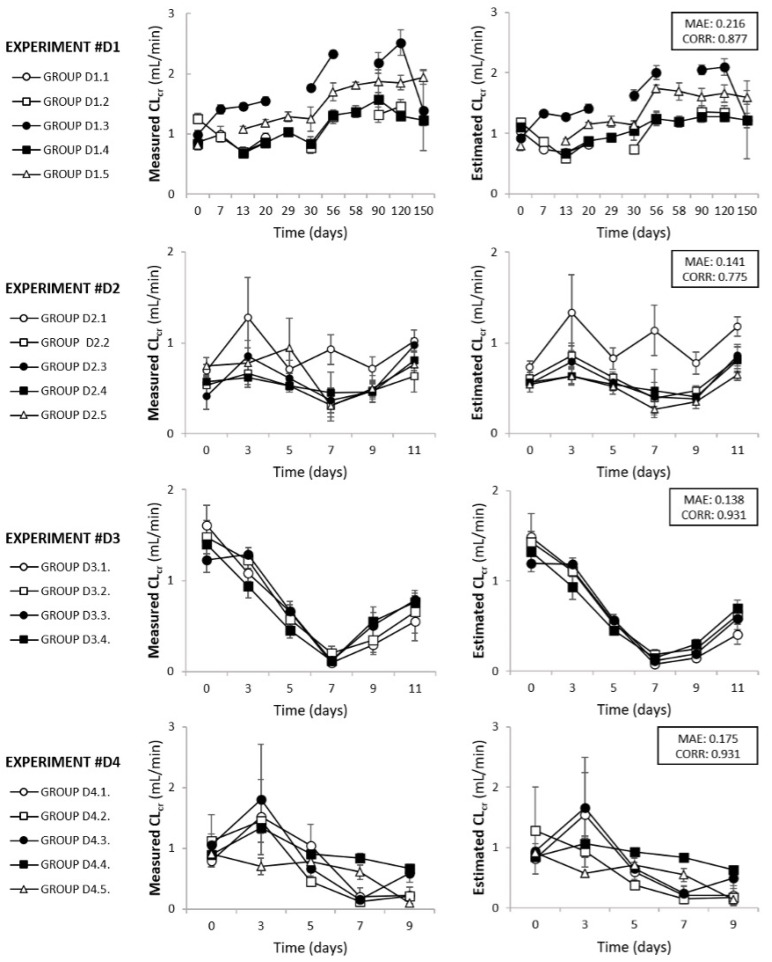
Comparison of the measured ClCr and estimated ClCr, corresponding to four exemplifying actual experiments using data from the training and validation sets. ClCr, creatinine clearance. CORR, Pearson product-moment correlation coefficient. MAE, mean average error.

In addition to analyzing the global performance of the model, the results of eClCr were also compared with their corresponding mClCr values in the context of individual experiments. Figure 2 shows the mClCr and eClCr for four representative experiments (from the training and validation datasets). In general terms, the information provided by measured ClCr (mClCr) and eClCr is almost identical. In particular, in those cases in which mClCr and eClCr are less similar (e.g., groups D2.1 and D2.5 in Figure 2), eClCr is consistently more congruent within its context. Just as an example, the group D2.1 is a control, and groups D2.2 to D2.5 refer to animals treated with cisplatin in several conditions. There is no reason for mClCr to increase in group D2.5 on day 5. On the contrary, following a similar pattern to the other AKI groups, eClCr shows a more logical result for group D2.5 and overall.

### 3.2. Model Field Testing

**Figure 3:**
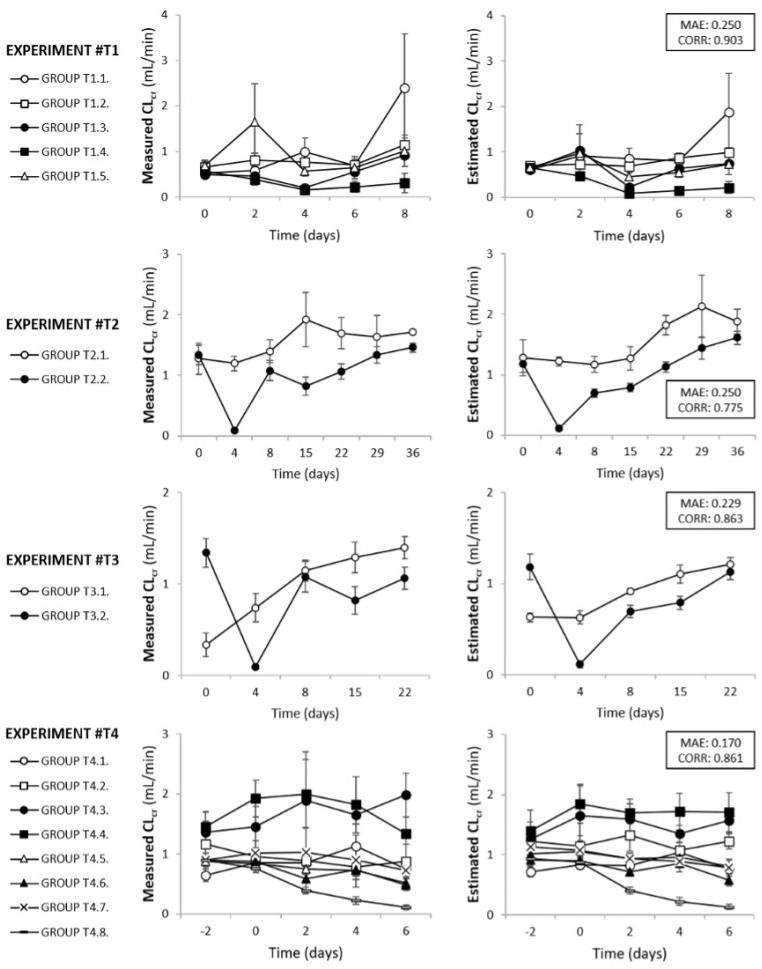
Comparison of the measured ClCr and estimated ClCr, corresponding to four exemplifying actual experiments, using data from the test set. ClCr, creatinine clearance. CORR, Pearson product-moment correlation coefficient. MAE, mean average error.

As shown in Table 2, the MAE and Pearson product-moment correlation coefficient for the test set were 0.2035 and 0.8564, respectively, which are similar to those obtained in the training and validation sets, hence proving that the model is not overfitted and can generalize to unseen data. Furthermore, a paired *t*-test was employed to compare the mean of the absolute error committed by the NN and the LR models in the test set; the NN was found to be superior with very high statistical significance (*p* < 0.001, using SciPy library [48], version 1.5.2). As above, eClCr predictions were compared with their corresponding mClCr values in the context of specific experiments. Figure 3 shows the mClCr and eClCr for four illustrative experiments (from the test data). There is a good match at the whole experiment level between mClCr and eClCr. Again, eClCr is more congruent than mClCr. As exemplifying tokens, the mClCr in group T1.5 by day 2 and group T1.1 by day 8 (a control) shows illogical increments that are normalized or softened by the corresponding eClCr. In experiment T2, the open circles correspond to the control group and the filled circles to a highly nephrotoxic, one-dose treatment. As expected, by day 4, there is a notorious drop in the GFR, which is progressively restored afterwards. The gradual recovery described by eClCr contrasts with the more erratic trajectory depicted by mClCr.

**Figure 4:**
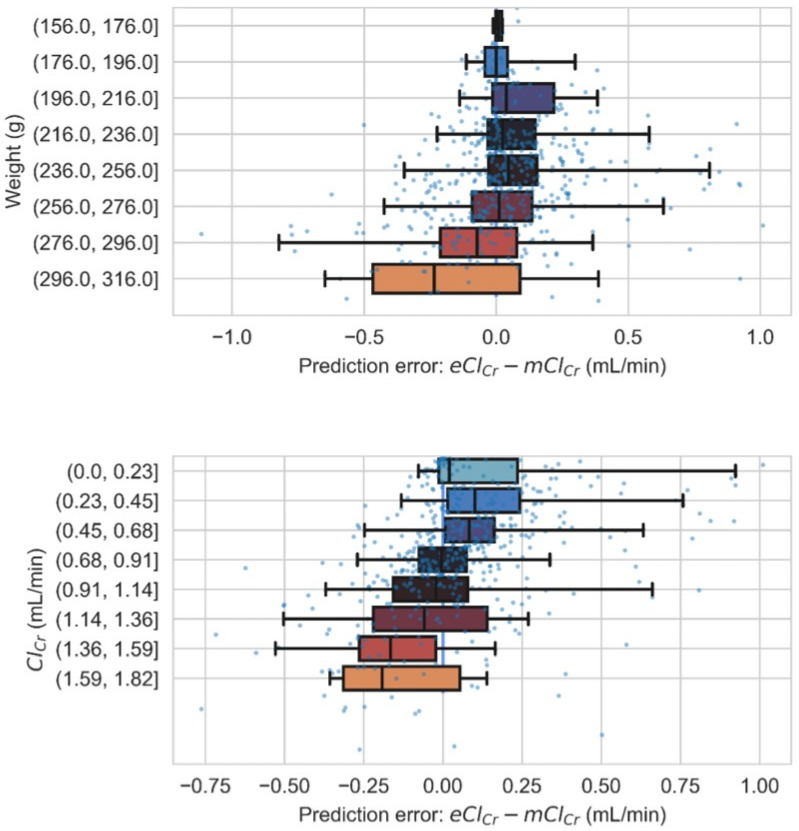
Prediction error analysis. Prediction error box plots according to the rat’s body weight range (**top**) and the mClCr range (**bottom**). Error bars represent the 2.5th and 97.5th percentiles. A scatter plot of prediction errors is also shown superimposed.

The influence of weight and mClCr on the distribution of the prediction error (eClCr-mClCr) in the whole test set is shown in Figure 4. The variance of the prediction error grows (top panel) with the weight, probably as a result of an increasingly lower amount of data for higher weights. The medians suggest that the model may underestimate the ClCr for high weights. In contrast, the variance of the prediction error seems to be similar for all ClCr values (bottom panel). Yet the medians show that the model tends to slightly overestimate ClCr for small to medium mClCr values (below 0.683 mL/min) and to slightly underestimate ClCr for medium to larger mClCr values (above 1.138 mL/min).

## 4. Discussion

Our study reveals that an FFNN fed with pCr and weight values as inputs predicts ClCr (i.e., eClCr) with reasonably high accuracy, regardless of additional variables such as age, comorbidities, or treatment. This calculator may be useful for the estimation of ClCr in those laboratories where metabolic cages are not available, or when avoidance of experimental complexity and resource optimization are sought. 

When evaluated with the training, validation, and test datasets, the model showed a relatively low average error. Furthermore, the model proved especially useful for evaluating experiments on the whole. In fact, the behavior and evolution of eClCr for each group was, in general, more homogeneous and congruent than the behavior of mClCr, while the relations among experimental groups resulting from eClCr remained extremely similar to those obtained with mClCr. Our interpretation of this data-equalization effect is that, as opposed to mClCr, eClCr obviates the error introduced by urine collection in metabolic cages, which is unavoidably translated to uCr estimation and, in turn, to mClCr, and thus sometimes yields unexplainable and illogical fluctuations (as argued in the Results section). This advantage adds to the procedural simplicity of eClCr compared to mClCr. Accordingly, this calculator is not aimed at being a method for the precise determination of ClCr but a practical tool providing rapid, easy, and sufficiently accurate results for many purposes of basic research with rats, in the same way that human GFR-estimating formulas are useful in the clinical setting. In our view, this calculator is particularly appropriate for those experiments in which it is not the exact individual value of ClCr that is required but rather its comparison among experimental groups or time points. In addition to facilitating preclinical research, eClCr also provides an alternative to mClCr that improves experimental animal welfare. Individual confinement in metabolic cages [37,38] required for urine collection is known to stress social animals such as rats [49]. Hence, this method is tightly aligned with the 3Rs principles (replacement, reduction, and refinement) concerning animal research [50].

In rats, pCr is mostly constant throughout their lifespan. However, the GFR (and thus ClCr) increases within an initial age range, after which it largely stabilizes [51]. Therefore, age should be taken into account when modeling the ClCr–pCr relationship. Yet because rat age might not be precisely known by the researcher in some cases, we used weight as a surrogate and found that its inclusion significantly improved model performance (even more than age itself). Furthermore, we contend that weight recapitulates other potential sources of disparity related to body habitus. Ethnicity and sex were not included in the model as we did not have sufficient data from other rat strains, nor of female rats. Accordingly, this model is adjusted only for male Wistar rats. Its performance must also be tested, and the model adjusted (if and as necessary), for different rat strains and female sex.

Another important factor potentially uncoupling the relationship between pCr and ClCr is creatinine secretion. Differential affecting of creatinine secretion by different experimental conditions might increase variability and hamper model performance. However, the model was generated on heterogeneous data from etiologically diverse disease models, and there were no deviation tendencies to specific characteristics. This implies that creatinine secretion is not significantly modified by the treatments or conditions included and that the observed data variability mainly reflects experimental variability derived from sample collection and analytic error.

## 5. Conclusions

The proposed model is a reasonably accurate surrogate for mClCr, solves some of its faults, and even provides more congruent results. eGFR in humans is known to be less accurate in specific populations and circumstances, such as individuals with abnormal muscle mass or body surface area; during changes in metabolism and pregnancy; and when GFR is rapidly changing, such as during growth, AKI, or high-protein ingestion [16,52]. In contrast, laboratory rats present a lower phenotypic heterogeneity as compared to humans, so a higher accuracy is therefore expected. Lastly, we have released ACLARA, a web-based, openly available eClCr calculator (http://idal.uv.es/aclara, accessed on 5 February 2022), to facilitate its use and encourage further research on the topic.

## Figures and Tables

**Table 1:** Characteristics of the datasets used for model development and field testing. CTRL, control. T-AKI, toxic acute kidney injury. I/R, ischemia/reperfusion. M, male. RMR, 5/6 renal mass reduction. PRD, predisposed-to-AKI (as in [39,40,41,42]).

Model	n (Rats)	Age at Start (Weeks)	Duration (Weeks)	n (Data Trios)
Model Development (Training + Validation Datasets)				
CTRL	9	8	1	51
	34	8	2–28	194
T-AKI	109	8	1	583
	19	8	2–8	98
	64	8	2–28	381
I/R	15	8	2–8	63
RMR	35	8	2–28	184
Test Dataset				
CTRL	13	8	1	59
	2	8	5	10
	3	8	7	21
PRD	61	8	1	279
T-AKI	9	8	5	38
	7	8	7	49
	50	8	1	204

**Table 2:** Performance metrics for the three considered models in the training and validation sets. LR, linear regression. RF, random forest. FFNN, feed forward neural network. MAE, mean average error. Correlation, Pearson product-moment correlation coefficient. P10/P30, fraction of predictions with an error within the 10%/30% threshold. Best results for each set are highlighted in bold.

	Training Set			Validation Set			Test Set		
	LR	RF	FFNN	LR	RF	FFNN	LR	RF	FFNN
MAE	0.2210	**0.1801**	0.1956	0.1975	0.1894	**0.1780**	0.2515	0.2257	**0.2035**
Correlation	0.8402	**0.8869**	0.8632	0.8732	0.8871	**0.9028**	0.7922	0.7450	**0.8563**
P10	0.2635	**0.3322**	0.3099	0.2699	0.2828	**0.2879**	0.2530	0.2803	**0.3136**
P30	0.6901	**0.7751**	0.7391	0.7044	0.7189	**0.7532**	0.6394	0.6727	**0.6955**

## Data Availability

Data will be available upon reasonable request.

## Supplementary Materials

The following supporting information can be downloaded at: https://www.mdpi.com/article/10.3390/biomedicines10030610/s1, Figure S1: Data transformation. Figure S2: Graphical description of the FFNN architecture. Figure S3: FFNN decision surface map. Figure S4: Bland–Altman plot of agreement between eClCr and mClCr in the test set.
