# Supplementary material for: Neural Network-Based Calculator for Rat Glomerular Filtration Rate
Source: Biomedicines. 2022 Mar 5;10(3):610. doi: 10.3390/biomedicines10030610 (PMC8945373; doi:10.3390/biomedicines10030610)
Supplement: Supplementary file 1 [file biomedicines-10-00610-s001.zip › biomedicines-1607292-supplementary.pdf]

Article

Neural Network-Based Calculator For Rat Glomerular Filtration Rate

Óscar J. Pellicer-Valero [1,†], Giampiero A. Massaro [2,3,4,5,6,†], Alfredo G. Casanova [2,3,4,5,6], María Paniagua-Sancho [2,3,4,5,6], Isabel Fuentes-Calvo [2,3,5,6], Mykola Harvat [1], José D. Martín-Guerrero [1,7,‡], Carlos Martínez-Salgado [2,3,5,6,7,‡] and Francisco J. López-Hernández [2,3,4,5,6,7,8,*,‡]

[1] Intelligent Data Analysis Laboratory (IDAL), Department Electronic Engineering, School of Engineering (ETSE-UV), Universitat de València, 46100 Valencia, Spain; oscar.pellicer@uv.es (Ó.J.P.-V.); mykola.harvat@uv.es (M.H.); jose.d.martin@uv.es (J.D.M.-G.)
[2] Institute of Biomedical Research of Salamanca, 37007 Salamanca, Spain; giampieroandrea.massaro@usal.es (G.A.M.); alfredogcp@usal.es (A.G.C.); meripani@usal.es (M.P.-S.); ifc@usal.es (I.F.-C.); carlosms@usal.es (C.M.-S.)
[3] Departmento de Fisiología y Farmacología, Universidad de Salamanca, 37007 Salamanca, Spain
[4] Fundación Instituto de Estudios de Ciencias de la Salud de Castilla y León, 42002 Soria, Spain
[5] Group of Translational Research on Renal and Cardiovascular Diseases (TRECARD), 37007 Salamanca, Spain
[6] National Network for Kidney Research REDINREN, RD016/0009/0025, Instituto de Salud Carlos III,
28029 Madrid, Spain
[7] Disease and Theranostic Modelling Consortium (DisMOD), 37007 Salamanca, Spain
[8] Group of Biomedical Research on Critical Care (BioCritic), 47003 Valladolid, Spain
* Correspondence: flopezher@usal.es
† These authors share first authorship.
‡ These authors share senior authorship.

Supplementary Material

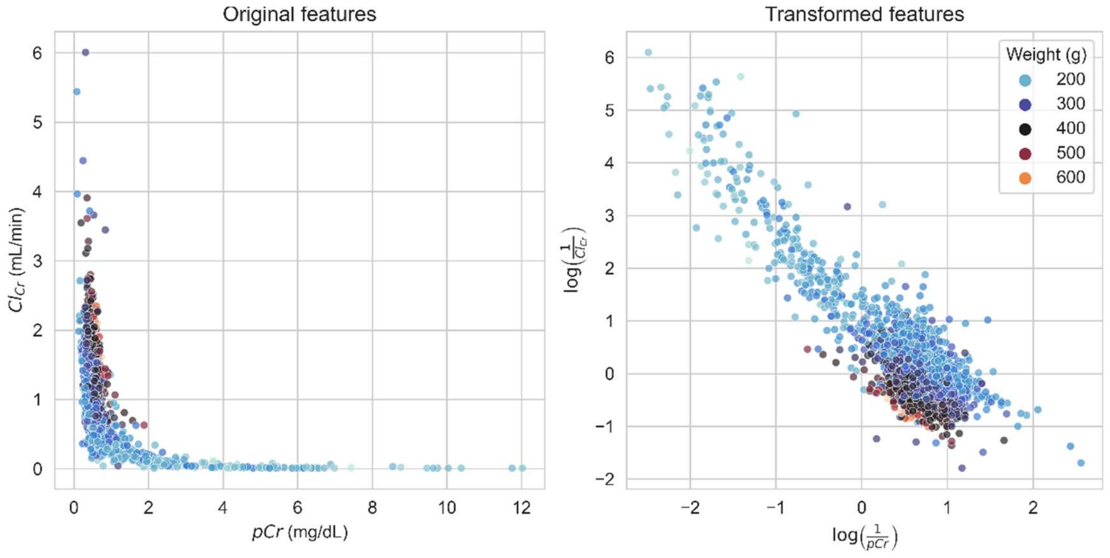

Figure S1. Data transformation. Scatter plots of the original creatinine clearance (Cl_{Cr}) and plasma creatinine concentration (pCr) data (left), and the data transformed by Equation 2 (right), color-coded by weight.

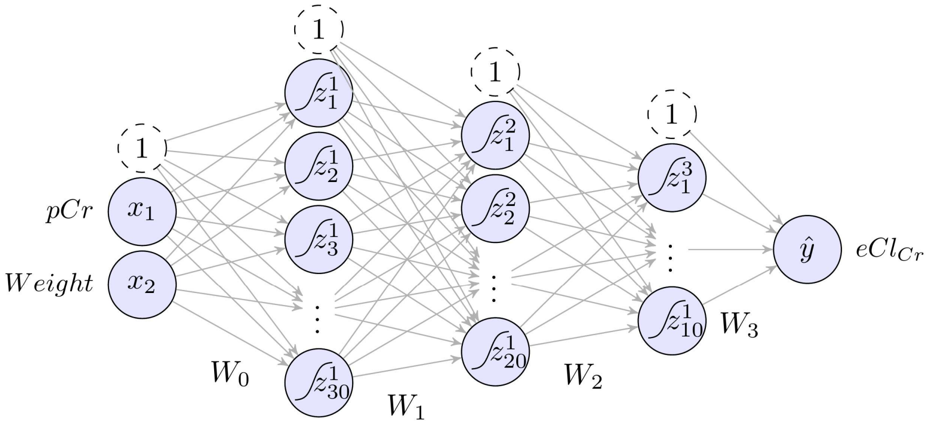

Figure S2. Graphical description of the FFNN architecture. Input features (pCr and weight) along with a bias feature (always taking a value of 1) were fed through a series of layers until an estimated ClCr (eClCr) was obtained as the output. Neurons in each layer (i.e. colored circles), had a value of z obtained as a linear projection (i.e. a LR) of all the values from the previous layer and then passed through a non-linear activation function. The linear projection is defined by a matrix of W parameters (i.e. weights) (e.g., from layer 1 to layer 2: $z^2 = W_1 \cdot z^1$), whose values are graphically represented as arrows. The hyperbolic tangent (tanh) was used as the non-linear activation function, and it is denoted by an S-shaped curve present in intermediate layer neurons.

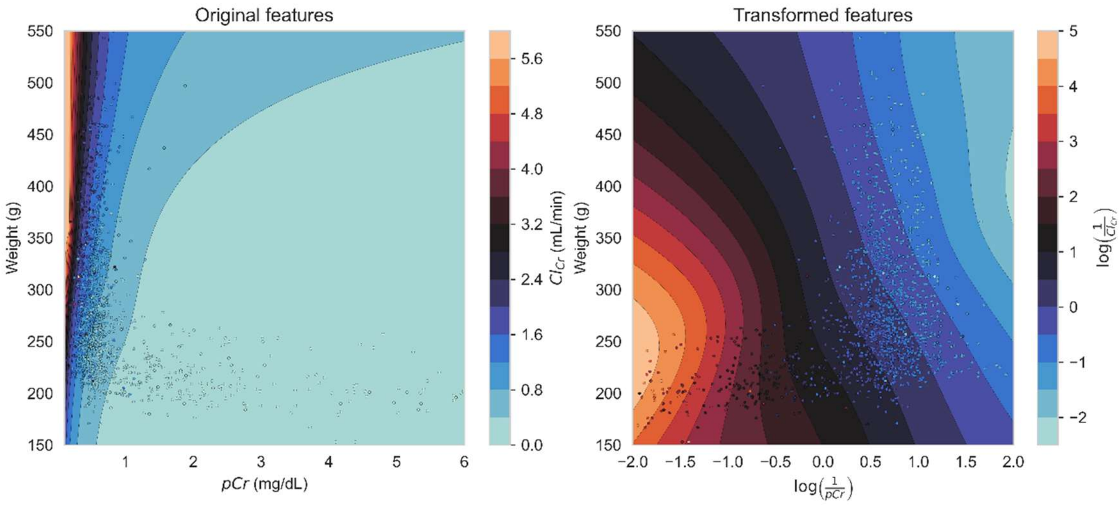

Figure S3. FFNN decision surface map, as evaluated on the original features (left) and the transformed features (right). The background colors represent the output of the model for all possible input values. Actual data points are added with colors corresponding to their values, using the same scale as for the background. Therefore, the closer the color of a point to the corresponding background color, the better the match between the measured and predicted value. This representation defines completely what the model has learned and the influence that each input feature has on the output. ClCr, creatinine clearance. pCr, plasma creatinine concentration.

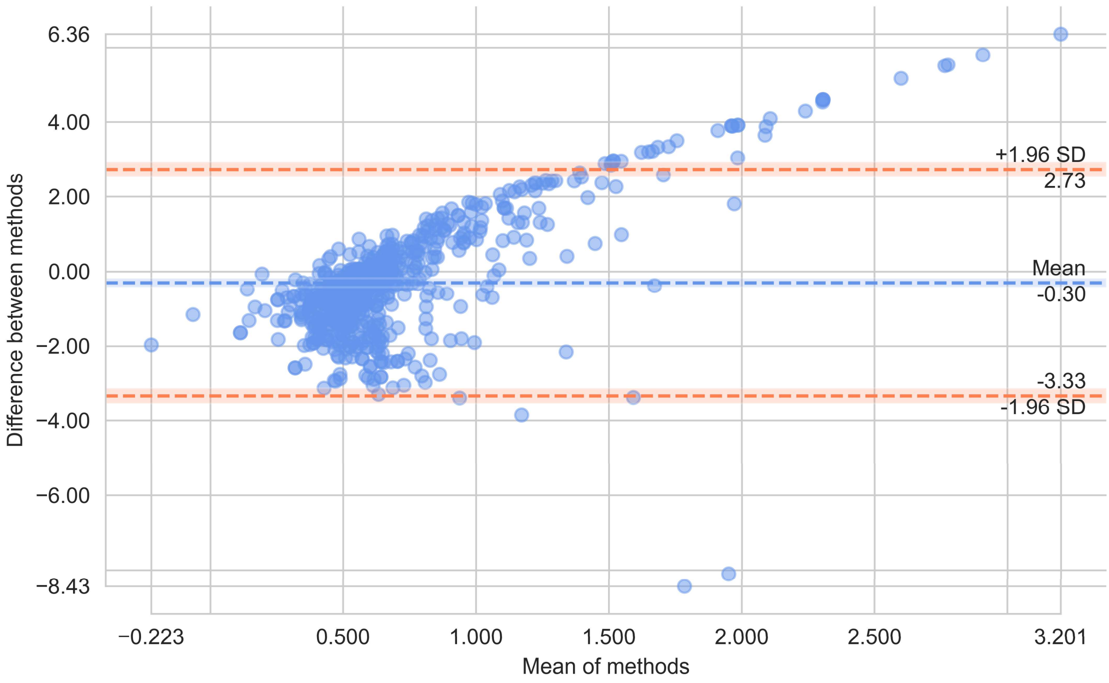

Figure S4. Bland-Altman plot of agreement between eCl_{Cr} and mCl_{Cr} in the test set.
